# Leaf Extracts of *Calocedrus formosana* (Florin) Induce G2/M Cell Cycle Arrest and Apoptosis in Human Bladder Cancer Cells

**DOI:** 10.1155/2011/380923

**Published:** 2011-06-12

**Authors:** Sheau-Yun Yuan, Chi-Chen Lin, Shih-Lan Hsu, Ya-Wen Cheng, Jyh-Horng Wu, Chen-Li Cheng, Chi-Rei Yang

**Affiliations:** ^1^Institute of Medicine, Chung Shan Medical University, Taichung 40201, Taiwan; ^2^Division of Urology, Department of Surgery, Taichung Veterans General Hospital, Taichung 40705, Taiwan; ^3^Department of Education and Research, Taichung Veterans General Hospital, Taichung 40705, Taiwan; ^4^Institute of Biomedical Science, National Chung-Hsing University, Taichung 40227, Taiwan; ^5^Department of Forestry, National Chung-Hsing University, Taichung 40227, Taiwan

## Abstract

*Calocedrus formosana* (Florin) bark acetone/ethylacetate extracts are known to exert an antitumor effect on some human cancer cell lines, but the mechanism is yet to be defined. The aim of this study was to determine the effects of Florin leaf methanol extracts on the growth and apoptosis of human bladder cancer cell lines. MTT (3-(4,5-Dimethylthiazol-2-yl)-2,5-diphenyltetrazolium bromide) assay showed that the growth of these bladder cancer cells was potently inhibited by the Florin leaf extracts. The cell cycle of these extract-treated cells (TCCSUP cells) was arrested at the G2/M phase as determined by flow cytometry. Western blot analysis revealed the increases of cyclin B1 and Cdc2 kinase levels, alone with the decrease of phosphorylated Cdc2 kinase, after treating these cells with the extracts. An immunofluorescence assessment of **β**-tubulin showed decreased levels of polymerized tubulin in treated cells. However, the proteolytic cleavage of poly ADP-ribose polymerase and the activation of caspase-3/-8/-9 were all increased upon treatments of extracts. The concurrent increase of Bax and decrease of Bcl-2 levels indicated that the extracts could induce apoptosis in these treated cells. Taken together, these results suggest that the Florin leaf extracts may be an effective antibladder cancer agent.

## 1. Introduction

According to a recent study, urinary bladder cancer including transitional cell carcinoma (TCC) affects more than 2 million people worldwide [1]. Bladder cancer has been ranked as the seventh most common type of cancer in Taiwanese males and is still rising in incidence and prevalence [2]. Usually, bladder cancers are grouped into two types of clinical manifestation, depending on the pathological stage: low-grade (G1, G2)/noninvasive cancers (pTa, pT1) and high-grade (G3)/muscle invasive lesions (greater than pT2) [3]. Intermediate to high-grade or metastatic TCC is more difficult to treat and is lethal in ~50% of patients [1].

Recently, many natural products have served as pharmaceutical resources in treating and preventing human diseases throughout the world [4, 5]. For example, over 60% of prevalent anticancer drugs, including vinblastine, topotecan, etoposide, and paclitaxel, were originally plant-derived compounds [6, 7]. *Calocedrus formosana* (Florin) belongs to the *Cupressaceae* family and grows at an altitude of 800–1500 meters in the north central mountain region of Taiwan [8]. Extracts from the bark of Florin have been suggested to possess some bio-active effects, such as anti-oxidative [9], anti-inflammatory [10], immunoregulatory [11], antitermitic, and antifungal activities [8, 12]. It has also been proposed to have antitumoral properties [13]. Most studies have focused on the Florin bark as a medical source, but few studies have investigated the use of the Florin leaf as an anticancer pharmaceutical resource. In this study, we investigated the effect of Florin leaf methanol extracts on the growth of human bladder carcinoma cells, including TCCSUP cells that are derived from a high-grade and invasive human urinary bladder tumor [14]. Here we demonstrate that the Florin leaf methanol extracts inhibit growth of these bladder carcinoma cells by arresting cell cycle at the G2/M phase and inducing apoptosis.

## 2. Materials and Methods

### 2.1. Preparation of Florin Extracts

The Florin leaves were collected from the Hui-Sun Forest Station of National Chung Hsing University in Taichung, Taiwan. Leaves were washed, air-dried, and extracted twice with methanol by ultrasonication for 30 min at room temperature. The extracts were then filtered, concentrated, and then lyophilized. Florin extracts was prepared by dissolving the lyophilized powder in dimethylsulfoxide to a final concentration of 50 mg/mL. The stock was stored at −20°C until use.

### 2.2. Cell Culture

Human bladder cancer cell lines (TCCSUP, T24, TSGH-8301, and RT4 cells) and SV-40-immortalized normal uroepithelial cells (SV-HUC-1 cells) were purchased from the Food Industry Research and Development Institute (FIRDI) (Hsinchu, Taiwan). TCCSUP cell line (Grade IV, mutant p53) was isolated from an anaplastic transitional cell carcinoma (TCC) [14]; T24 cells were derived from an invasive bladder tumor of grade III, having *p53 *nonsense mutation at codon 126 (TAC to TAG); TSGH-8301 cells (grade II), having wt *p53 *but mutant *Rb *gene, were derived from a well-differentiated human TCC; RT4 cells (grade I) were established from a well-differentiated papillary tumor of the bladder and have the wt *p53 *and *Rb *gene [15]. Cell lines were cultured in McCoy's 5A and RPMI medium supplemented with 10% fetal bovine serum (FBS) (Gibco, Gaithersburg, MD), L-glutamine (200 mM), and penicillin/streptomycin/amphotericin B (10,000 IU/mL, 10,000 *μ*g/mL, and 25 *μ*g/mL) solution. Cells were incubated at 37°C with 5% CO_2_. 

### 2.3. Cell Survival Assay

Bladder cancer cells and normal uroepithelial cells (1 × 10^4^) were plated onto 24-well plates and treated with Florin extracts at concentrations of 3, 6, 12, 25, and 50 *μ*g/mL or vehicle alone for 48 h. MTT (3-(4,5-Dimethylthiazol-2-yl)-2,5-diphenyltetrazolium bromide) solution (200 *μ*L from 1 mg/mL) was added to each well, and the plates were further incubated at 37°C for 4 h. The medium was aspirated and the formazan product in cells was solubilized by adding DMSO. An aliquot of 150 *μ*L was measured by a Microplate Autoreader (Tecan Deutschland GmbH, Crailsheim, Germany) at wavelength of 570 nm. The experiments were carried out in triplicate.

### 2.4. Apoptosis Assay-Annexin V Apoptosis and DAPI Staining

Florin-extract treated TCCSUP cells were stained by FITC-conjugated Annexin-V and propidium iodide (PI) using an Annexin V-FITC Apoptosis Detection kit (BioVision, CA, USA) and analyzed by a Becton-Dickinson FACSCalibur with CellQuest software (BD Biosciences, San Diego, CA, USA). After 24 h of treatment, the cells were washed with PBS and fixed in 2% paraformaldehyde for 30 min, and then permeabilized with 0.1% Triton-X 100 in PBS for 30 min. Nuclei were stained by incubating the cells with DAPI (1 *μ*g/mL) and examined under a fluorescence microscope. Five randomly-chosen fields of view per well were inspected and the number of intact nuclei and the number of multinuclear cells were counted.

### 2.5. Cell Cycle Distribution by Flow Cytometry Analysis

The treated cells were collected after trypsinization and washed with ice-cold PBS, fixed and permeabilized with 70% ethanol at −20°C overnight. On the next day, after cells were washed with ice-cold PBS, they were incubated with PI (20 *μ*g/mL) and RNase (100 *μ*g/mL) for 30 min at room temperature in the dark. Data were collected from the flow cytometer and analyzed with the accompanying software (CellQuest; BD Biosciences, San Diego, CA, USA). Ten thousand events per sample were counted and the experiments were performed in triplicate. Data represent the means ± standard deviations of 3 independent experiments.

### 2.6. Western Blot Analysis

Cell lysates with equal amounts of proteins, which were measured using a BCA Protein Reagent Kit (Pierce, Rockford, IL, USA), were analyzed by Western blot, using a rabbit polyclonal antibody to cdc2 phosphorylated at Tyr15 (p-Cdc2) (1 : 1000; R&D, Minneapolis, MN, USA), a rabbit polyclonal cdc2 antibody (1 : 1000; Cell Signaling Technology, St. Louis, MO, USA), a mouse monoclonal antibody to cyclin B1 (sc-254; 1 : 200) (Santa Cruz Biotechnology, Santa Cruz, CA, USA), a mouse monoclonal antibody to poly (ADP-ribose) polymerase (PARP) (0.5 *μ*g/mL; from BD Biosciences, Franklin Lakes, NJ, USA), a mouse monoclonal antibody to Bcl-2, a rabbit polyclonal antibody to Bax (1 : 200; both from DAKO, Taipei, Taiwan), an anti-*β*-tubulin mouse monoclonal antibody (1 : 20000; Epitomics, Burlingame, California), or a mouse monoclonal antibody against human GAPDH (1 : 1000; Santa Cruz, CA, USA). Briefly, samples were run on 12% sodium dodecylsulfate (SDS)-polyacrylamide gels and electrophoretically transferred (SDS-PAGE) to nitrocellulose membranes (Bio-Rad Laboratories, Hercules, CA). Nonspecific binding sites were blocked with 5% skim milk powder diluted in PBS with 0.1% Tween 20 (SMP/PBST). Membranes were reacted with primary antibody followed by incubation with horseradish peroxidase-linked goat antirabbit or goat antimouse secondary antibodies (Santa Cruz, CA, USA) which were also diluted in PBST. Proteins were visualized by enhanced chemiluminescence (Amersham Biosciences, San Francisco, CA, USA). Each blot was stripped with Restore Western Blot Stripping Buffer (Millipore, Billerica, MA, USA) before being reprobed with other antibodies.

### 2.7. Morphological Observation of Cells and Immunofluorescence Confocal Microscopy for Tubulin

TCCSUP cells (1 × 10^4^) were treated by Florin extracts for 24 h. The cell morphology was observed under a reverse microscope. For tubulin analysis, cells (1 × 10^4^) were first seeded onto 2-well chamber slides (Lab-Tek^R^; Nunc, Roskilde, Denmark) and treated Florin extracts (25 *μ*g/mL) for 24 h. Cells were prefixed with 0.5% glutaraldehyde in 0.25% Triton X-100 in cytoskeleton buffer (CB) for 60 s, washed twice (15 min each) with CB, and refixed with 1% glutaraldehyde in CB for 10 min. The slides were blocked with 2% BSA in PBST (0.1% Tween 20 in PBS) for 2 h and reacted with anti-*β*-tubulin rabbit monoclonal antibody (Abcam, Cambridge, MA, USA) at 4°C overnight. Slides were washed three times with PBST and incubated with Alexa 488-conjugated antirabbit IgG rabbit antibody (Invitrogen, Carlsbad, CA, USA) for 1 h at room temperature. Cells were counterstained with DAPI (1 *μ*g/mL) and examined using a ZEISS LSM 510 confocal spectral microscope.

### 2.8. Analysis of Soluble and Polymerized *β*-Tubulin

The cells (2 × 10^6^) were precultured for 24 h and treated with Florin extracts (25 *μ*g/mL), colchicine (0.05 *μ*g/mL), or Taxol (0.1 *μ*g/mL) for 24 h. After the cells were harvested with a scraper and washed with PBS, the cells were added to 100 *μ*L of microtubule-stabilizing buffer (MSB: 20 mM Tris-HCl, 1 mM MgCl_2_, 2 mM EGTA, and 0.5% Triton X-100) containing a proteinase inhibitor cocktail (Roche, Indianapolis, IN, USA). After incubation for 20 min at room temperature, the cell suspensions were centrifuged at 12,000 × g for 5 min at 25°C. The supernatants (soluble fraction) were transferred to a new tube, while pellets were washed with MSB once and mixed with 50 *μ*L of protein lysis buffer (RIPA). After incubation at 4°C for 20 min, the supernatants (polymerized fraction) were harvested by centrifugation at 100,000 × g for 30 min at 4°C. Soluble and polymerized fractions were mixed separately with 4X NuPAGE LDS sample buffer (Invitrogen) and stored until analysis. Twenty micrograms of soluble and polymerized fractions were subjected to 10% SDS-polyacrylamide (SDS-PAGE) gel electrophoresis. The separated proteins on SDS-PAGE gel were electrically transferred to a PVDF membrane (Perkin Elmer, Boston, MA, USA) for immunoblot analysis. The membrane was incubated with anti-*β*-tubulin rabbit monoclonal antibody (1 : 20,000; Abcam, Cambridge, USA) 4°C overnight and then incubated with horseradish peroxidase-labeled antirabbit secondary antibody (1 : 5000, R&D, Minneapolis, MN, USA) for 1 h. The membrane was developed using the ECL plus Western blot detection system (Amersham Biosciences, San Francisco, CA, USA).

### 2.9. Determination of Caspase Activity

We used the colorimetric substrates Ac-DEVD-pNA, Ac-IETD-pNA, and Ac-LEHD-pNA (Biovision, California, USA) for caspase-3, -8, and -9 assays, respectively, according to the manufacturer's protocol. Briefly, aliquots of cell lysates were prepared in lysis buffer (50 mM HEPES, pH 7.4, 100 mM NaCl, 0.1% CHAPS, 1 mM dithiothreitol, 0.1 mM EDTA) and then incubated with 200 *μ*M of substrate in assay buffer (50 mM HEPES, pH 7.4, 100 mM NaCl, 0.1% CHAPS, 10 mM dithiothreitol, 0.1 mM EDTA, 10% glycerol) in 96-well plates at 37°C for 2 h. Absorbance of the cleaved product was measured at 405 nm in a TECAN Microplate Reader.

### 2.10. Statistics

All data are presented as mean ± S.D. The differences between the treated cells and the control cells were analyzed by Student's *t*-test. A *P *value <  .05 was considered statistically significant.

## 3. Results

### 3.1. Inhibition of Bladder Cancer Cell Growth by Different Dosages of Florin Extracts

**Figure 1:**
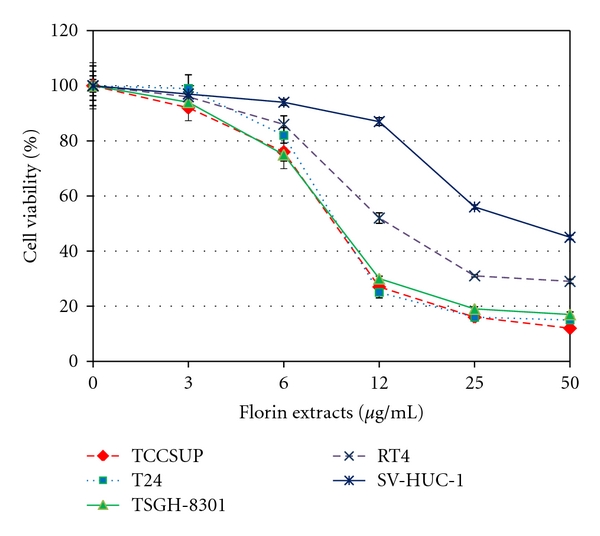
Effects of Florin extracts on cell viability of human bladder cancer cells (TCCSUP, T-24, TSGH-8301, and RT-4 cells) and immortalized normal uroepithelial cells (SV-HUC-1 cells). These cells were treated with various concentrations (3, 6, 12, 25, and 50 *μ*g/mL) of Florin extracts or vehicle only for 48 h. Results are presented as mean ± S.D. (*n* = 3).

Four bladder cancer cell lines (including TCCSUP, T24, TSGH-8301, and RT-4 cells) and immortalized normal uroepithelial cells (SV-HUC-1) were treated with various concentrations (3–50 *μ*g/mL) of Florin extracts for 48 h. The growth of these cells was determined by MTT assay. As shown in Figure 1, treatment with Florin extracts for 48 h inhibited growth of T-24, TCCSUP & TSGH-8301 cells, and RT-4 cells in a concentration-dependent manner with IC_50_s of ~9-10 and ~17 *μ*g/mL, respectively. Treatment of SV-immortalized normal uroepithelial cells (SV-HUC-1 cells) with Florin extracts for 48 h also exhibited concentration-dependent growth inhibition but with a higher IC_50_ (~27 *μ*g/mL). These results indicate that Florin extracts inhibit growth of malignant bladder cancer cells more effectively than normal uroepithelial cells. 

### 3.2. Apoptotic Effect of Florin Extracts on Bladder Cancer Cells

**Figure 2:**
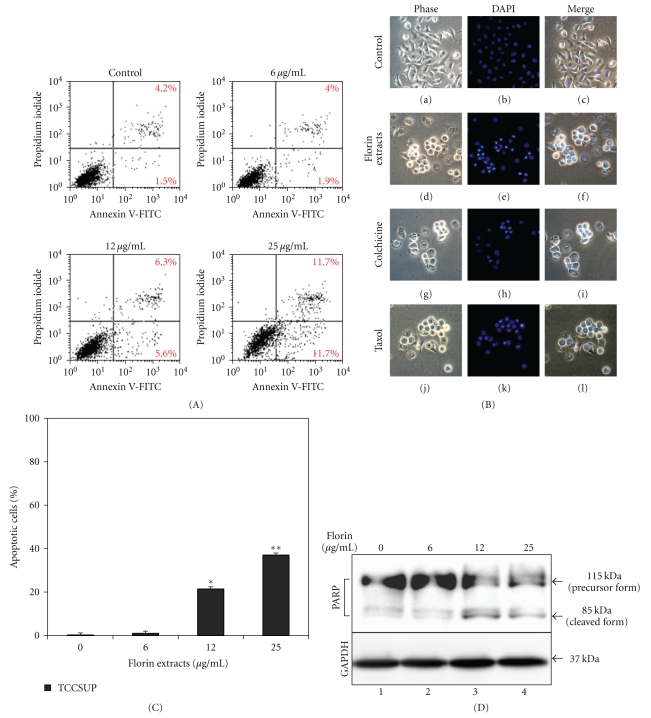
Analysis of the effect of Florin extracts on apoptosis in TCCSUP cells. (A) Cells were treated with 0, 6, 12, or 25 *μ*g/mL of Florin extracts. After 18 h, the percentage of early apoptotic cells was assessed by Annexin-V/PI binding assay. (B) After 24 h, the adherent cells or suspension cells were collected and subjected to DAPI staining. (C) The ratio of DAPI-stained cells was calculated and the data are presented as mean ± S.D. *P < .05, **P < .01. (D) After the cells were treated with 0, 6, 12, and 25 *μ*g/mL of Florin extracts for 48 h, the PARP protein was resolved on 10% SDS-PAGE and then Western blot was performed. GAPDH was used as a control.

TCCSUP cells were treated with 25 *μ*g/mL of Florin extracts for 18 and 24 h, and then stained with Annexin V-FITC and DAPI. With Annexin V-FITC staining, early apoptosis was clearly detectable in cells treated with 25 *μ*g/mL of Florin extracts for 18 h (11.7%) (Figure 2(A)). The apoptotic nucleus-containing adherent and supernatant cells, which exhibited highly fluorescent condensed chromatin and cleaved nuclei, were observed in cells treated with 25 *μ*g/mL of Florin extracts (Figure 2(B), e and f). The apoptosis index of these treated cells is shown in Figure 2(C). After 48 h of treatment with 12 *μ*g/mL or 25 *μ*g/mL of Florin extracts, a 85-kDa proteolytic product of PARP was observed (Figure 2(D), lanes 3 and 4). 

### 3.3. Cell Cycle Arrest at the G2/M Phase

**Figure 3:**
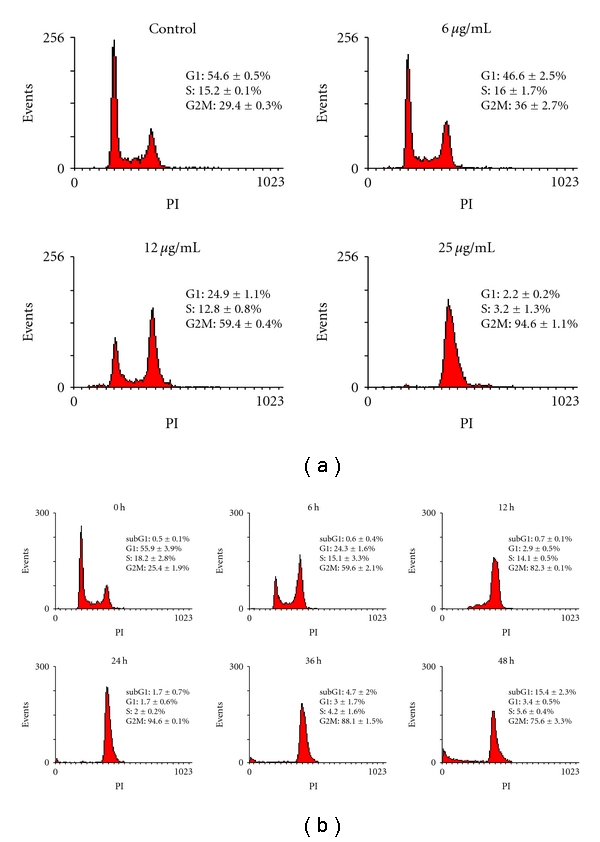
Flow cytometry analysis of TCCSUP cells treated with Florin extracts. (a) Cells were treated with 6, 12, and 25 *μ*g/mL of Florin extract for 24 h. (b) Cells were treated with 25 *μ*g/mL of Florin extracts for 6, 12, 24, 36, and 48 h and then stained with PI. Florin extracts induced significant cell cycle arrest at the G2/M phase; however, cell numbers decreased significantly at phase G1 (*P < .05).

To study the effect of Florin extracts on the different phases of cell cycles, we mainly used TCCSUP cells for the experiments. TCCSUP cells were treated with various concentrations (6, 12, and 25 *μ*g/mL) of Florin extracts for 24 h and then analyzed by a fluorescence-activated cell sorter (FACS). The percentage of TCCSUP cells at the G2/M phase markedly increased in a Florin-extracts concentration-dependent manner (Figure 3(a)). The percentages of TCCSUP cells in the G2/M phase were estimated to be 29.4 ± 0.3, 36.0 ± 2.7, 59.4 ± 0.4, and 94.6 ± 1.1% after treatment of these cells with 0, 6, 12, and 25 *μ*g/mL of Florin extracts, respectively (Table 1). These results indicate that Florin extracts arrest TCCSUP cells at the G2/M phase. To test the time dependence of the effect, cells were treated with 25 *μ*g/mL of Florin extracts for 6, 12, 24, 36, and 48 h, and the cell cycle of treated cells were analyzed. As shown in Figure 3(b) and Table 2, the G2/M arrest was observed at 6 h (59.6 ± 2.1%), reached a maximal level (>90%) (94.6 ± 0.1%) at 24 h, and then gradually decreased (due to cell death). The sub-G1 cells increased to 15.4 ± 2.3% at 48 h. These results suggest that Florin extracts induce G2/M phase arrest in a time-dependent manner. 

### 3.4. Effects of Florin Extracts on Cell Cycle-Related and Apoptotic Proteins

**Figure 4:**
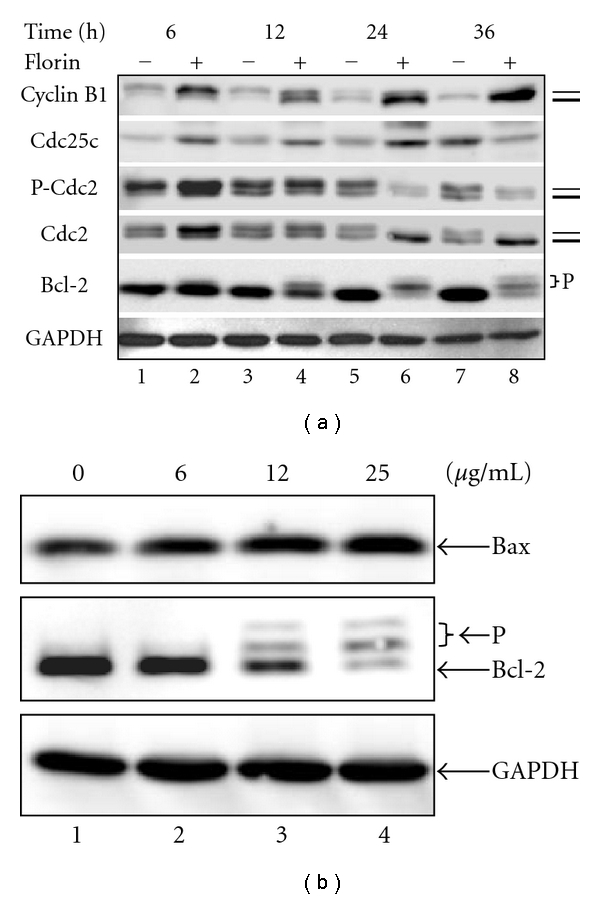
Western blot analysis of cell cycle regulatory protein levels and apoptosis proteins in TCCSUP cells treated with Florin extracts. (a) Cdc25C, Cdc-2, pCdc-2, cyclin-B1, and Bcl-2 proteins were analyzed after cells were exposed to Florin extracts. Cells were treated with 25 *μ*g/mL of Florin extracts for 6, 12, 24, and 36 h. Proteins (50 *μ*g) from each sample was resolved on 12% SDS-PAGE and Western blot was performed. GAPDH was used as a control. The thick and thin bars indicate the locations of the fast (dephosphorylated) and slow (phosphorylated) migrating forms of cyclin B1, P-Cdc2, or Cdc2, respectively. (b) Expression of Bcl-2 and Bax proteins in TCCSUP cells was analyzed. Cells were treated with 6, 12, and 25 *μ*g/mL of Florin extracts for 24 h. Proteins (50 *μ*g) from each sample were resolved on 12% SDS-PAGE and Western blot was performed. GAPDH was used as a control. The two slow migrating forms of Bcl2 may be the hyperphosphorylated forms of Bcl2.

The expression of cyclin B1, Cdc2, and phosphorylated Cdc2 (p-Cdc2), key regulators of cell entry into mitosis, was monitored by immunoblotting assay. The treatment time points are as indicated in Figure 3(b). As shown in Figure 4(a), the levels of cyclin B1 and Cdc2 kinase were upregulated after a 6 h treatment of TCCSUP cells with Florin extracts (lane 2). Interestingly, the slow migrating forms of cyclin B1 and Cdc2 were converted to the fast migrating forms of these molecules after treatment of cells with Florin extracts for 24 and 36 h (lanes 6 and 8). The slow and fast migrating forms may represent hyperphosphorylated and hypophosphorylated (or dephosphorylated) forms of cyclin B1 and Cdc2, respectively. Cdc25C was upregulated after treatment of the cells with Florin extracts for 6, 12, and 24 h (Figure 4(a), lanes 2, 4, and 6), then decreased after 36 h treatment (lane 8), as compared with untreated cells (Figure 4(a), lanes 1, 3 and 5). On the other hand, Florin extracts induced a decrease of P-Cdc2 in a time-dependent manner. Because the Cdc2 level was not significantly altered after treatment of cells with Florin extracts for 12, 24, and 36 h, this suggests that treatment with Florin extracts induces dephosphorylation of P-Cdc2 in a time-dependent manner. This suggestion is supported by the observation that the hypophosphorylated or dephosphorylated form (fast migrating form) of Cdc2 was the major molecule of Cdc2 in cells treated with Florin extracts for 24 and 48 h (Figure 4(a), lanes 6 and 8). Increase of cyclin B1 levels and Cdc2 dephosphorylation (activation) is known to be associated with mitotic arrest [16, 17]. These results support the suggestion that Florin extracts arrest cells at the G2/M phase of the cell cycle. In this study, we also found that Florin-extracts induced phosphorylation (slow migrating form) and degradation of Bcl-2 correlated with the production of the fast migrating form (dephosphorylated form) of Cdc-2 (Figure 4(a), lanes 6 and 8). These results may imply that the increase of Cdc2 kinase activity is associated with degradation or instability of Bcl-2, an antiapoptotic protein. Furthermore, after TCCSUP cells were treated with 12 or 25 *μ*g/mL of Florin extracts for 24 h, the level of Bax, a proapoptotic protein, increased in accompany with the decrease of Bcl-2 levels (Figure 4(b), lanes 3 and 4). 

### 3.5. Florin Extracts Inhibit Tubulin Polymerization in TCCSUP Cells

**Figure 5:**
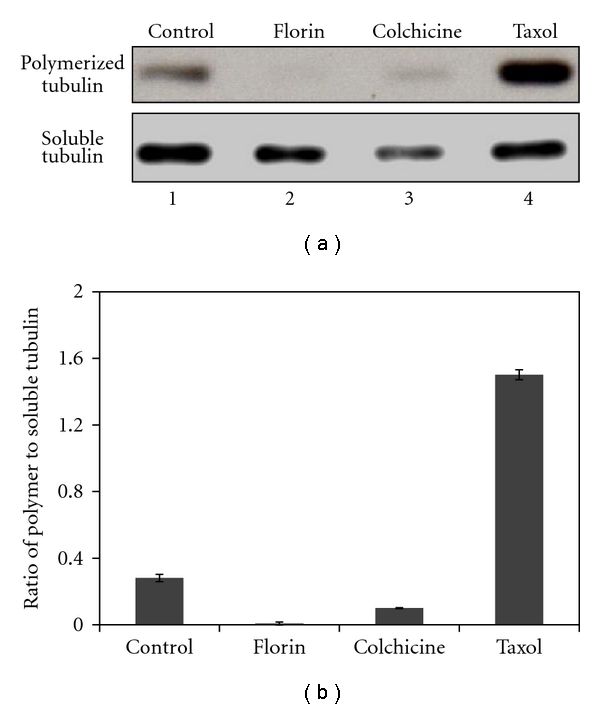
Determination of the percentages of polymerized to soluble *β*-tubulin in TCCSUP cells treated with Florin extracts. Cells (2 × 10^6^) were pre-cultured for 24 h and treated with Florin extracts (25 *μ*g/mL), colchicine (0.05 *μ*g/mL), or Taxol (0.1 *μ*g/mL) for 24 h. Treated cells were harvested, lyzed, and soluble and polymerized fractions of tubulin were obtained. About 25 *μ*g of soluble and polymerized fraction proteins were used in the Western blot analysis, with antirabbit *β*-tubulin monoclonal antibody as a primary antibody. Images were photographed (a), and the percentages of polymerized to soluble tubulin were calculated using the band area (b).

**Figure 6:**
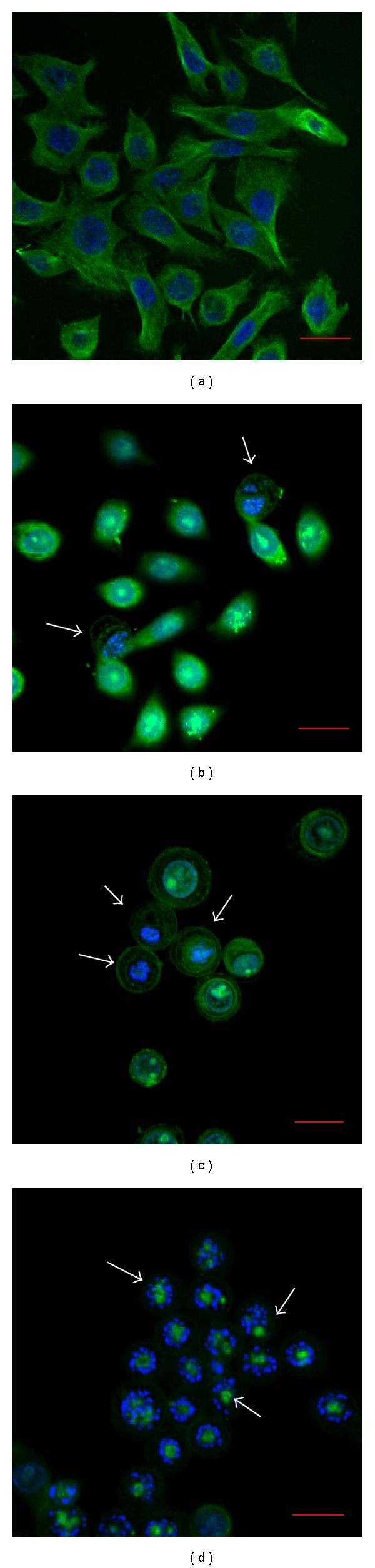
Effect of treatment with Florin extracts on the amount of prometaphase microtubules in TCCSUP cells. (a) Cells were incubated with vehicle (0.1% dimethylsulfoxide) for 24 h. (b) Cells were treated with 25 *μ*g/mL of Florin extracts, (c) 0.1 *μ*g/mL of colchicine, or (d) 0.1 *μ*g/mL Taxol for 24 h. Control indicates vehicle-treated cells. Microtubules (green) and nuclei (blue) are shown. Scale bar, 20 *μ*m. Some of the *β*-tubulin are indicated by white arrows in (b), (c), and (d). (630x original magnification).

We next wanted to determine whether the quantitative change in tubulin levels and the altered balance of polymerization and depolymerization account for the effects of Florin extracts. We fractioned the soluble and polymerized tubulin in Florin-extract-treated cells, performed Western blot analysis, and determined the percentage of tubulin polymers to monomers. As shown in Figure 5(a), treatment of cells with Florin extracts for 24 h dramatically decreased the tubulin levels (>90%) as compared to control. Treatment with Taxol (0.1 *μ*g/mL), an inhibitor of tubulin depolymerization, profoundly increased the polymerized tubulin (approximately fivefold as compared to control) (Figure 5(b)). We also determined the effects of Florin extracts on the microtubule network in TCCSUP cells by immunofluorescence confocal microscopy (Figure 6). Normal prometaphase microtubule organization in the control cells is shown in Figure 6(a). Treatment with a high concentration of Florin (25 *μ*g/mL) caused a significant reduction of microtubule density in the apoptotic cells (Figure 6(b)). Furthermore, treatment with colchicine (0.1 *μ*g/mL) resulted in inhibition of microtubule polymerization and reduction of microtubule density in the cytoplasm (Figure 6(c)). In contrast, Taxol (0.1 *μ*g/mL) treatment resulted in the formation of microtubule “bundles” (Figure 6(d)). In the experiments, we also determined that the amount of *β*-tubulin which represented half of the total amount of polymerized tubulin. (data not shown).

### 3.6. Effects of Florin Extracts on the Activities of Caspase-3, -8, and -9 in TCCSUP Cells

**Figure 7:**
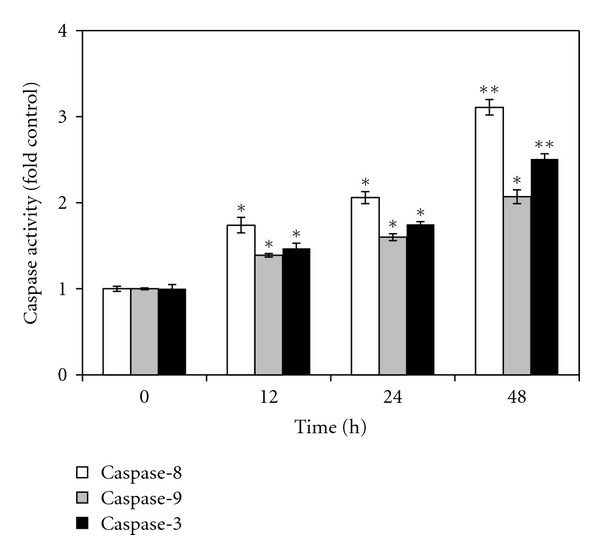
Effects of Florin extracts on caspase-3, -8, and -9 activities in TCCSUP cells. After treatment with 25 *μ*g/mL of Florin extracts for 12, 24, and 48 h, cell lysates were prepared and enzymatic activities of caspase-3, -8, and -9 were measured by colorimetric assay. The caspase-3, -8, and -9 activities in control cells were taken as 1-fold, and the relative changes of these activities in the treated cells were estimated (*P < .05, **P < .01).

Caspase-3, -8, and -9 play crucial roles in the apoptotic pathway, which contain a mitochondria-related “intrinsic pathway” and death receptor-related “extrinsic pathway” [18, 19]. To study the effect of time length on caspase-3, -8, and -9 activities, TCCSUP cells were treated with 25 *μ*g/mL of Florin extracts for 12, 24, and 48 h. The activities of these enzymes in the treated cells were analyzed. As shown in Figure 7, compared to the control treatment, caspase-8 and -3 activities were significantly increased after treatment of cells with Florin extracts for 12, 24, and 48 h. For example, the caspase-9 activity was increased by 1.5-fold after 24 h, as compared to the control treatment. 

## 4. Discussion

In the current study, we have demonstrated that Florin extracts inhibited cell proliferation (Figure 1), and arrested the cell growth at the G2/M phase (Figure 3). Florin extracts appeared to have a potent selectivity toward 4 bladder cancer cells (IC_50_s = ~9–17 *μ*g/mL versus IC_50_ = ~27 *μ*g/mL in SV40-immortalized normal uroepithelial cells). Presently, Florin extracts used is very crude; its antibladder cancer cell activity with IC_50_s of 9–17 *μ*g/mL is relatively potent. However, it is possible that the blood or tissue concentrations of Florin extracts active components may not be able to reach the IC_50_s. To address this potential problem, one could increase the dosage of Florin extracts to a few hundred mg/kg. However, the potential side or toxicity effects of this high dose of Florin extracts have to be addressed. Alternatively, the active components in the Florin extracts could be further concentrated or purified by conventional chromatographic techniques using antibladder cancer cell activity assay. Further studies may also be required to identify the active components responsible for the observed antitumor effect of Florin extracts. 

 Upregulation of cyclin B1/Cdc2 kinase activity is known to be involved in the G2/M phase transition of the cell cycle [20, 21]. Cyclin B1 and Cdc2 kinase regulate the entry and progression of the mitotic phase in eukaryotic cells. A previous report showed that the activation of Cdc2 kinase at the G2/M transition requires accumulation of cyclin B1 and Cdc2 [17, 21]. Cdc2 kinase is activated by the specific phosphatase Cdc25C [22, 23]. Here we demonstrate that treatment of bladder cancer cells with Florin extracts results in an increase of cyclin B1/Cdc2 kinase activity as evidenced by increased levels of dephosphorylated Cdc2 kinase (activated Cdc2 kinase) in cells treated with Florin extracts. The microtubule network plays an important role in the regulation of mitotic apparatus. Cells are arrested in the M phase when microtubule dynamics are disrupted. Our data shows that treatment with Florin extracts induces depolymerization of microtubules in bladder cancer cells. This occurs by decreasing *β*-tubulin expression in these cells. Microtubules are composed of *α*-tubulin and *β*-tubulin. Several reports have indicated that both the microtubule-stabilizing agents (such as Taxol) and the microtubule-depolymerizing agents (such as Vinblastine and colchicine) [24–26] exhibit potent anticancer activity by inhibiting cell cycle progression and inducing cell apoptosis [26, 27]. Thus, we used colchicine and Taxol as controls. The experimental results reveal that treatment of bladder cancer cells with Florin extracts leads to cell cycle arrest at the G2/M phase. This appears to be mediated, at least in part, by depolymerizing microtubules as colchicine does. 

Anti-microtubule agents, including Taxol, Doxetaxel, Vincristine, and colchicine, have been shown to induce cell growth arrest, phosphorylation of Bcl-2, increased Bax protein levels, and finally cell death. This involves the mitochondrial pathway and activation of caspase-9 and caspase-3 [28–30]. Bcl-2 is known to play an important role in the intrinsic apoptosis pathway and protects the microtubule integrity [18]. Here we demonstrated that treatment with Florin extracts induce apoptosis in bladder cancer cells, as determined by Annexin-V assay and caspase-3, -8, and -9 activity measurement. The apoptotic activity of Florin extracts in bladder cancer cells appears to be mediated by (1) increase of cyclin B1/Cdc2 kinase activity, (2) inhibition of tubulin polymerization, (3) phosphorylation and degradation of Bcl-2, an antiapoptotic protein, and (4) increased production of Bax, an apoptotic protein. 

The death receptor-signaling cascade belongs to the extrinsic pathway [19] which involves activation of caspase-8. Our study shows that TCCSUP cells treated with Florin for 12 h exhibit up-regulation of both initiator caspase (caspase-8 and -9) and effector caspase (caspase-3) and that caspase-8 is upregulated earlier than caspase-9 and caspase-3 in these cells treated with Florin extracts. Caspase-3, which cleaves PARP protein, plays an important role in the execution of apoptosis. Elevated levels of caspase-3 (up to 2.5-fold of control) are observed after 48 h of Florin-extract treatment. Therefore, our results suggest that apoptosis of Florin extract-treated TCCSUP cells occurs through the arrest of the cell cycle at the G2/M phase and then proceeds through both the death receptor and the mitochondrial pathways. 

## Figures and Tables

**Table 1:** Flow cytometry analysis in TCCSUP cells treated with Florin extracts.

Dose	Percentage (mean ± S.D.) of TCCSUP bladder cancer cells at each cell cycle phase after treatment with different concentrations of Florin extracts (*μ*g/mL) at 24 h.			
	subG1 (%)	G1 (%)	S (%)	G2/M (%)

0 (control)	0.8 ± 0.3	54.6 ± 0.5	15.2 ± 0.1	29.4 ± 0.3
6	1.4 ± 0.5	46.6 ± 2.5	16.0 ± 1.7	36.0 ± 2.7*
12	2.9 ± 0.1	24.9 ± 1.1	12.8 ± 0.8	59.4 ± 0.4**
25	1.3 ± 0.2	2.2 ± 0.2	1.9 ± 1.3	94.6 ± 1.1***

^a^TCCSUP cell growth was arrested at the G2/M phase after Florin-extract treatment in a dose-dependent manner, and these cells were significantly different from the control cells.

^b^Cell numbers decreased significantly at the G1 phase (*P < .05, **P < .01, ***P < .001).

**Table 2:** Flow cytometry analysis in TCCSUP cells treated with Florin extracts.

Time (h)	Percentage (mean ± S.D.) of TCCSUP bladder cancer cells at each cell cycle phase after treatment with Florin extract (25 *μ*g/mL) at different time points			
	subG1 (%)	G1 (%)	S (%)	G2M (%)

0	0.5 ± 0.1	55.9 ± 3.9	18.2 ± 2.8	25.4 ± 1.9
6	0.6 ± 0.4	24.3 ± 1.6	15.1 ± 3.3	59.6 ± 2.1**
12	0.7 ± 0.1	2.9 ± 0.5	14.1 ± 0.5	82.3 ± 0.1***
24	1.7 ± 0.7	1.7 ± 0.6	2.0 ± 0.2	94.6 ± 0.1***
36	4.7 ± 2.0	3.0 ± 1.7	4.2 ± 1.6	88.1 ± 1.5***
48	15.4 ± 2.3	3.4 ± 0.5	5.6 ± 0.4	75.6 ± 3.3***

^a^TCCSUP cell growth was arrested at the G2/M phase after Florin-extracts treatment in a time-dependent manner, and these cells were significantly different from those at baseline (0).

^b^There was a significant difference between these cells and those in the control group (**P < .01, ***P < .001).
